# Bibliography

**Published:** 1886-03

**Authors:** 


### BIBLIOGRAPHY.

### BOOKS AND PAMPHLETS RECEIVED.

*A Compend of the Practice of Medicine*—Physician's edition based on the revision of the Quiz-Compend edition, including a very complete section on skin diseases, by Daniel E. Hughes, M. D., Demonstrator of Clinical Medicine in the Jefferson Medical College, Philadelphia; Fellow of the College of Physicians of Philadelphia, etc., pp. 399. P. Blakiston, Son & Co., Philadelphia.

This book says more in a small space than any work now out. It is well arranged, well written, and comes right up to date. The busy practitioner cannot do without it. The reputation of the author is a sufficient guarantee of the work's merits.

We note that the *Medical Analect*, published by G. P. Putnam's Sons, has passed into the editorial management of Dr. R. W. Amidon.

The *Kansas City Medical Index* comes to us for January, entirely changed in shape, appearance and style, and greatly improved in every way, and enlarged to 100 pages. The improvement shows the enterprise, pluck and ability of those gentlemen now at the editorial helm, Drs. Elston and Lanphear. We wish them great success. See club rates.

---

How to Use Listerine.—A man may have a fine microscope and it will be useless, without some knowledge of how to work it to advantage. So—Mrs. Hale found it necessary to tell people how to cook a hare; a hare or a turkey would be perfectly useless unless we know what to do with it. So with Listerine. True, anybody could sprinkle it on; but Mr. Lambert, the benefactor, has done another benefaction by telling physicians *how to use* Listerine to get its best results, both internally and externally. But, as Mrs. Hale began her directions about cooking the hare with—"fiirst; get the hare,"—we suppose the first step in the matter in hand will be to procure a supply of Listerine, (and here we may remark that no physician who pretends to be up with modern surgery and practice can afford to dispense with this valuable deodorizer, and antiseptic), for, experience has demonstrated it to be the best and safest antiseptic for internal use in all zymotic and febrile diseases, as well as in wounds and ulcers, etc. The book gives minute directions. Sent free on application. Mention this journal.

---

*The Prairie Queen*, a monthly magazine for the family circle. $2.00 a year, single copies 20 cents. Edited by Mrs. W. B. Brooks, Mrs. J. H. Richardson and Miss Nannie E. Burts, Fort Worth, Texas; Prairie Queen Publishing Co.

We have received vol. 1, No. 1 of this new candidate for favor in the literary world, and it looks to have made a fair start, judging from the favorable press notices we have seen. It is gotten up in a very neat, modest style, and the table of contents embraces a pleasing variety of miscellaneous reading, romances, poetry, fashion notes, domestic receipts, etc., such as are usually found in family magazines. We extend to our young friend the right hand, and wish them success. It does look as if a literary magazine of a high order would meet with encouragement at the hands of the Texas public, but somehow all such efforts, so far as we know, have failed for want of it. Better luck to the *Prairie Queen.*

---

*Practical Human Anatomy*, by Fanuell D. Weisse, M. D., Prosector (1863 to 1865) to the late Valentine Mott, M. D., LL. D., Emeritus Professor of Surgery and Surgical Anatomy Medical Department of the University of the City of New York; Professor of Anatomy N. Y. College of Dentistry. Illustrated by 222 colored plates, and containing 321 figures. William Wood & Co., 56 and 58 Lafayette place, New York, 1886.

This work is especially designed as a working guide for students, and a ready reference for surgeons, and is admirably adapted to the purpose. It is much fuller than Gray's which for years has been the standard and which was thought to be about as near perfect as a work of the kind could be made. But there is one deficiency in Gray's which is well filled in the admirable work before us—the anatomy of the perineum. The dissections of the female perineum alone are worth to a surgeon the price of the book. Dr. Weisse has followed Gray's general plan, but has simplified many of the branches and divisions, and the book in its entirety, surpasses in point of practical utility anything ever before offered the profession.

The student of anatomy is taught all about the dissecting instruments, and the several steps in the progressive dissection of each part, and taught how to see and study each tissue involved in dissection; and is guided by lines showing where incisions are to be made for their reflection in order to advance to a succeeding stage, each part being numbered, and their "relatiods" clearly shown. The illustrations of the anatomy of the regions and viscera of the body, are well executed plates with the names of the parts printed upon them in clear type, so that as a book of reference a practitioner can use it to advantage without much study or search. In our judgment, this work is a desideratum to the surgeon's library long needed. The mechanical execution of the work is in a high degree artistic, and is a good specimen of Wood & Co. manufacture.

---

*Diseases of the Ear:* The Diagnosis and Treatment of, by Owen D. Pomeroy, M. D., Surgeon to the Eye and Ear Hospital;

Opthalmic and Aural Surgeon to the New York Infant Asylum; Consulting Surgeon to the Paterson Eye and Ear Infirmary; Member of the American Ophthalmological and Otological Societies, et., with ten illustrations, second edition, revised, with additions. New York, 1886: D. Appleton & Co.. 1, 3 and 5, Bond street; pp, 408. Cloth.

This book is designed, it seems, especially for the use of the general practitioner, and not for the specialist, and, as such, is admirably adapted to his wants. It is very elementary—beginning at the beginning, and therefore adapted to the beginner as well as the general practitioner. The domain of medicine is so broad and rapidly widening, that the impossibility of a thorough cultivation of each and every department by one man is generally recognized, yet such are the surroundings of most physicians that they are *compelled* to know *something* of the more advanced ideas on every possible subject, In the country a practitioner cannot send his ear cases to an aurist and his eye cases to an oculist; he must treat them himself, and if he does not know how, he gets badly left; he *must* know how, and that is what this book is for, with reference to ears—to teach him how to diagnose and treat its various diseases. Every country practitioner and those in small towns—in fact, every doctor who wants to treat ear diseases—would be better prepared by having read Dr. Pomeroy's very excellent text book. As to the execution of the work, Appleton & Co. know how to turn out books for neatness and for wear. Send for it.

---

*Electricity in Medicine*, practical suggestions respecting the varieties of electric currents, and the uses of electricity in medicine, with hints relating to the selection and care of electrical apparatus, by Ambrose L. Ranney, M. D., professor of Anatomy and Physiology of the Nervous System in the New York Post Graduate Medical School and Hospital, etc., etc. New York: D. Appleton & Co., 1, 3 and 5 Bond St., 1885; cloth, p. 147; price $1.00.

This valuable little work is offered the profession as a guide to the study of electricity in disease; it is the A. B. C. of the subject, as it were, for one cannot comprehend a treatise on the diagnosis and treatment of nervous diseases without a knowledge of electricity and electrical apparatus. This little book is therefore a brief summary of such information as the talented and experienced author who has *for years* been instructing graduates in the use of electricity in medical practice, deemed most essential to the proper use of that potent agent in practice.

